# Human capital and poverty reduction in OPEC member-countries

**DOI:** 10.1016/j.heliyon.2019.e02279

**Published:** 2019-08-23

**Authors:** Bosede Comfort Olopade, Henry Okodua, Muyiwa Oladosun, Abiola John Asaleye

**Affiliations:** a Department of Economics and Development Studies, College of Business and Social Sciences, Covenant University, Ota, Nigeria; b Department of Economics, College of Business and Social Sciences, Landmark University, Nigeria

**Keywords:** Economics, Societal welfare, Human capital, OPEC, Poverty reduction

## Abstract

A vast study has shown a mixed result on the implications of a natural resource on growth and poverty. Theoretically, the Resource Curse Hypothesis stresses that natural resource serves as an obstacle for growth. However, the connection between human capital and poverty in OPEC member countries remain under-researched. To ensure inclusiveness in growth, it is essential to focus on human capital models that incorporate the components of poverty reduction. As a result, this study investigates the interactive relationship between human capital components and poverty reduction in OPEC member countries. It is a cross-country study of a panel fully modified least-squares of 12 countries within the OPEC region. The interactive effects of the components of human capital development have a long-run impact on poverty reduction in OPEC member countries. Besides, human capital components confirm a positive effect on poverty reduction. Thus, since human capital is a crucial determinant of improving economic growth, OPEC member countries should invest more on the quality of human capital through education and health to improve the living standard of people and societal welfare.

## 1 Introduction

There has been a noticeable improvement in global economic performance in the last two decades, both in absolute and relative terms due to development in human capital formation. For instance, global output increased from 31.3 trillion United State dollar (USD) in 1996 to 73.4 USD trillion in 2015; having an increase of 134.4 per cent. However, official statistics have shown weak interaction between human capital through education and health on poverty in the OPEC member countries. For example, the fall in oil price in 2015 led to a fall in the total output of oil in all member countries, resulting in a recession in some OPEC member countries, Nigeria and Venezuela inclusive. Although, the recession was not only traceable to the reduction in price but due to continuous attacks on pipelines in Nigeria. Likewise, in Venezuela, the ongoing political and economic crises have reduced production, leading to a cash-crunch and other shortages. Similarly, high level of unemployment and income inequality were found prevalent in most OPEC member countries despite the endowments in natural resources (Muzima, 2018; Messkoub, 2008; Popoola et al., 2018; IMF, 2014; Asaleye et al., 2018; ANND, 2009; Fosu, 2017; Asaleye et al., 2019). Evidence from the official statistics showed that in spite of the growth performance in Algeria, Nigeria, Iran and Iraq before the reduction in oil price in 2016, poverty and other features of non-inclusive growth remained very high (World Development Indicators, WDI, 2016; Oloni et al., 2017). The poverty rates in countries like Nigeria, Angola, Algeria and Ecuador were more than 20 per cent over the years. On the contrary, Iraq and Venezuela maintained a very lower poverty rate (less than 6 per cent) over the same period (WDI, 2016).

In the same vein, the Educational attainment in OPEC member countries has varied over the years. In 2010, the secondary school enrollment rates were as low as 29 per cent and 44 per cent in Angola and Nigeria respectively. On the contrary, Algeria, Ecuador, Saudi Arabia and Qatar have exceeded 100 per cent gross enrollment rate (World Bank, 2016). Likewise, evidence of health status in OPEC member countries, using the infant mortality rate as a pointer shows that Angola and Nigeria recorded significant changes during the period in the last decade, the rates are still very high in other OPEC member countries especially in Sub-Sahara countries (World Bank, 2016). Concerning investment in education, the Governments of Iran and Saudi Arabia spent more than 20 per cent (on the average) of their annual expenditure to develop the education sector in the last decade. On the other hand, Angola's spending as a percentage of total government expenditure on health was less than 10 per cent throughout the period (World Bank, 2016). Globally, total health expenditure per capita in 2014 ranged between US$ 9673 for Switzerland and 13 USD for Madagascar (World Bank 2016), the range is between 99 USD for Indonesia and 2106 USD for Qatar.

Evidence from both theoretical and empirical studies have shown mixed result on the implications of a natural resource on growth and poverty (Apergis and Katsaiti, 2018; Sachs and Warner, 1997; among others). On a negative side, a strand of literature shared the perspective that natural resource, most notably energy resource, does not reduce poverty; instead increases the poverty rate (Bulte et al., 2005; Ross, 2003; Goderis and Malone, 2011; Asaleye et al., 2018). A recent study by Apergis and Katsaiti (2018) investigated the relationship between poverty and resource curse using a global panel of countries. The scholars reported that energy resources worsen poverty across countries. Similarly, the Resource Curse Hypothesis emphasised that natural resource serves as an obstacle for growth (Sachs and Warner, 1995). While scholars like Papyrakis and Gerlagh (2004), Bravo-Ortega and De Gregorio (2005) pointed out that low rate of investment in human capital is one of the factors responsible for the Resource Curse Hypothesis. Consequently, Gylfason, Herbertsson and Zoega (1999), and Gylfason (2000) stressed that one of the major channels to transfer the natural resource to sustain growth and development is through human capital. More so, Goderis and Malone (2011) developed a model to investigate the nexus among human capital, inequality and natural resources. It was reported by the scholars that human capital plays a critical factor in the presence of natural resource to promote sustainable growth.

A significant strand of theoretical literature has stressed that there is a strong interactive effect between human capital and poverty. Human capital formation promotes economic benefits such as equality in distribution of income, enhance productivity and reduce unemployment rate (Becker, 1975; Santos, 2009; Silva and Sumarto, 2014; Fisher, 1946; Schultz, 1962; Teixeira, 2014; Roemer, 1998; World Bank, 2005). Empirically, Becker (1995) shows that there is a connection between human capital and poverty. According to the scholar, human capital has promoted sustainable growth in Japan, Taiwan, Hong Kong and South Korea despite the inadequacy of natural resources in those countries. Subsequently, the official statistics showed that government expenditure on education in OPEC countries in the last decade is less than 12 per cent on the average, although numerous improvement noted in the Republic of Iran and Saudi Arabia (UNESCO, 2018). Human capital development is essential for poverty reduction. Ensuring significant decrease in poverty is now a foremost objective of every economy, both developed and developing.

Based on the report by World Bank (2015), poverty is the deprivation in well-being and comprises of many dimensions. It includes low incomes and the inability to acquire the essential goods and services necessary for survival with dignity. It also encompasses low levels of health and education, poor access to clean water and sanitation, inadequate physical security, lack of voice, insufficient capacity and opportunity to improve one's life. Poverty reduction is regarded in this study as achieving economic growth through human capital development, which allows people to contribute to and benefit from economic growth. Subsequently, several factors that promote human capital development have been identified in the literature, and such factors include investment in human capital, job creation, structural transformation, entrepreneurship, social protection and institutions (Punam, 2014; Multidimensional Poverty Index Model, 2016; Cumming et al., 2019). However, one of the most notable is investments in human capital through lifelong learning (World Bank, 2015). The ‘Commission on Growth and Development’ (CGD, 2008) noted that human capital through education and health is a concept that promotes equality of opportunity and protection in the market and employment transition, which is an essential ingredient of any successful growth strategy. It maintains that investment in human capital will generate opportunities for growth, including opportunities unforeseen at the time of the investment. Human capital improves economic growth through its effect on total factor productivity. Apart from its role in enhancing overall factor productivity, the components of human capital (education and health) were found to have a positive effect on creation of equal opportunity for all citizens in the country (Mincer, 1991; Ridell and Song, 2011; Larionova and Varlamova, 2015).

Although numerous studies have investigated the implications of human capital on poverty. Few among others include the study by Attanasio et al. (2017) that examined the relationship between human capital growth and poverty in Ethiopia and Peru. Bhukuth, Roumane and Terrany (2018) that analyses the relationship between human capital and poverty. Also, Ucal and Bilgin (2009) examine the relationship between income inequality and FDI in turkey using fully modified OLS. However, few studies have examined the relationship between human capital and poverty in OPEC member countries despite the strong connection established in the literature. While many studies on poverty and human capital in these countries focused on a single country analysis. For example, Shahpari and Davoudi (2014) investigated the relationship between human capital and income inequality in Iran. Ogwumike and Ozughalu (2018) investigated the relationship between a child's poverty and deprivation in Nigeria, among others. The literature is replete with various analyses of its different components. Thus, theoretical and empirical studies on the following relationships exist in human capital and poverty (WHO, 2002); human capital and inequality (Blanden and Machin, 2004), human capital and unemployment (Arrow, 1973; Mincer, 1994); causality between entrepreneurship and poverty and its implications (Cumming et al., 2019), among others. This study aims to contribute to the literature by investigating the role of human capital on poverty reduction most especially in resources endowed countries given the implication of Resource Curse Hypothesis and the presumption of education to transform natural resource to sustain growth and development (Barro, 1997; Aghion et al., 1999; Marshall, 1920; Papyrakis and Gerlagh, 2004; Bravo-Ortega and De Gregorio, 2005).

Therefore, this study contributes to the existing literature is in two-fold. Firstly, most of the current studies using cross-sectional data focused on educational aspect only or either poverty and natural resource (Gamu et al., 2015; Loayza and Raddatz, 2010; Goderis and Malone, 2011; Fashina et al., 2018). This study includes health as one of the indicators used to proxy human capital. Although, many empirical studies have contributed in terms of measurement with poverty, both absolute and overall terms using different indexes, which varies. However, this index varies with the number of variables and the weights assigned to each indicator. Therefore, empirical findings will be highly subjective depending on the composition of both human capital and poverty index. This makes empirical findings biased in respect to the measurement and makes policy recommendation less inclusive. However, to ensure inclusiveness, it is essential for empirical studies on human capital development to focus on human capital models that incorporate the components of poverty reduction. Secondly, this study focused on OPEC member counties; this is necessary given the principle of ‘Resource Curse Hypothesis’. Low investment in human has been envisaged as one of the main factors resulting in the backwardness of most endowed economies. Thus, this study involves a cross-country analysis of a panel fully modified least-squares among the OPEC member countries. It is believed that this study will give insight on how health and education can be used to accelerate growth, increase income per capita and reduce poverty in OPEC members' countries.

In achieving this task, this paper streamlines into five sections after the introduction and brief literature review in Section 1. Section 2 is the model specification, while Section 3 is the estimation technique and sources of data. Section 4 is empirical results and discussion of findings. Section 5 is the summary of findings, policy recommendation and conclusion.

## 2 Material and method

To empirically model the relationship between human capital development and poverty reduction, the two-way interactive model is used to examine the impact of the interaction of human capital development on poverty reduction when public investment in education and health are below or above the global benchmark in selected OPEC member countries. However, to achieve this, multiple linear models that incorporate an interactive term is developed by using the multiplicative term between public investment in education and public expenditure on health. Conversely, this multiplicative term is represented on a scale, which predicts the effect of public investments in education and public investment in health and its impacts on poverty reduction in selected OPEC member countries. This study draws insights from the empirical works of Brambor et al. (2006) and Burrill (2003) in modelling and interpreting interactions in multiple regression using a linear model to represent the variations in a dependent variable as a linear function of several explanatory variables. In the same vein, Osabuobien and Efobi (2013) and Osoba and Tella (2017) used the interactive effect to address the nexus of human capital investment components and economic growth. Hence, this study introduced an interactive model to access the impact of human capital development on poverty reduction in selected OPEC member countries: Algeria, Angola, Congo Republic, Ecuador, Equatorial Guinea, Gabon, Republic of Iran, Kuwait, Libya, Nigeria, Saudi Arabia and Venezuela.

The baseline model for the objective of this study is presented in Eq. (1).

(1) $$pov_{it}=\alpha_{0}+\alpha_{1}educ_{it}+\alpha_{2}i.health_{it}+\alpha_{3}educ_{it}*i.health_{it}+\varepsilon_{it}$$

In Eq. (1), pov represent poverty rate as dependent variables, educ represent education, health represent health, educ∗health represent the interaction between education and health which are continuous variables,subsripti are entities). The i represents the dummy variables; $\alpha_{0}$ represents constant term; $\alpha_{1},\alpha_{2},\alpha_{3}$ represent parameters of the exogenous variables and ε represents the error term which measures other explanatory variables that are not explicitly captured in the model. Eq. (1) describes a multiple linear regression (MLR). However, taking into consideration the possible role that human capital development and poverty reduction interplay in the model, influencing government investment in both education and health, an interactive term is introduced, and this can be modified in an explicit form as:

(2) $$pov_{it}=\beta_{0}+\beta_{1}health_{it}+\beta_{2}i.educ_{it}+\beta_{3}health_{it}*i.educ_{it}+\varphi_{it}$$

In Eq. (2), ${\mathrm{Pov}}_{\mathrm{it}}$= Poverty rate as the dependent variable. $\beta_{o}$ = intercept of the equation. $\beta_{1}$$\beta_{2}$, $\beta_{3}$ represents parameters of the exogenous variables and φ represents the error term, which measures other explanatory variables that are not explicitly captured in the model. In MLR contexts, an interaction implies a change in the slope (of the regression of Poverty on investment in education and health) from one value of $\beta_{1}$ to another value of $\beta_{2}$ and the change of the slope is quantified by the value of $\beta_{3}$.

We progress by regressing poverty rate on the following four variables: dummy for education-adequate, the dummy for health-adequate, interaction between investments in education and dummy for health-adequate, as well as interaction between investments in health and dummy for education-adequate. This specified model applies to each of the OPEC member countries under investigation.

(3) $$pov_{it}=\gamma_{0}+\gamma_{1}i.educ_{it}+\gamma_{2}i.health_{it}+\gamma_{3}i.educ_{it}*health_{it}+\gamma_{4}i.health_{it}*educ_{it}+\mu_{it}$$

In Eq. (3), $\gamma_{0}$ represent a constant term, $\gamma_{1}i.educ_{i}represent$dummy for education, $\gamma_{2}i.health_{i}$ represent dummy for health, $\gamma_{3}i.educ_{i}*health_{i}$ represent the interaction between investments in health and dummy for education, $\gamma_{4}i.health_{i}*educ_{i}represent$interaction between investments in education and dummy for health $\gamma_{1}\text{, ​}\gamma_{2}\text{, ​}\gamma_{3}\text{ ​and ​}\gamma_{4}$ represents parameters of the exogenous variables and $\mu_{it}$is the error term.

The dependent variable in Eq. (1), which is the poverty rate, is measured by the headcount index of the international poverty line at 1.90 USD per day. The headcount measure is considered the most commonly calculated and used poverty measure. Education is proxied by government expenditure on education, while health is also proxied by government expenditure on health. Public investment in education and health are measured by human capital development index. Policymakers believed that human capital is a prerequisite for economic growth and also a key to reducing poverty, which has led virtually to the provision of investment in education and health in the developing world. However, for development to take place in developing countries, human capital has to be a mediator between growth and development. According to Bloom and Canning (2003) as quoted in Osoba and Tella (2017) researched that, one of the major ways of improving the quality of human resources and better productivity is through education and health services. The model assumes that education and health are crucial for economic growth. A positive relationship between public investment in education and health satisfies the condition for poverty reduction.

### 2.1 A priori expectation

The a priori expectation is such that: in Eq. (1) $\alpha_{1}$and $\alpha_{2}$< 0; in Eq. (2); β_1_, and β_2 <_ 0_;_ equation three γ_1_ and γ_2_ < 0; this means that an increase in public investment in health and increase in public investment in education have a reduction on the poverty rate. The sign of $\alpha_{3}$$\beta_{3}$ and γ_3_ cannot be inferred as a priori, as it depends on the nature of the respective interaction. In this regard, it means that if;

$\frac{\partial pov}{\partial \mathrm{int}eract\;educ*health}$ That is $\alpha_{3}$< 0, $\beta_{3}$ < 0 and γ_3_ < 0, it implies that public investment in education and health will reduce poverty. However, these two areas (education and health) have a complementary role in human capital development and poverty reduction in OPEC member states. The opposite holds if $\alpha_{3}$> 0, $\beta_{3}$ > 0 and γ_3_ > 0. Human capital investment in education and health will increase poverty. However, education and health as a variable assume either of two numerical values of ‘0' or ‘1' in which case they are treated as a dummy variable in this study. That is, education and health are assumed to reduce poverty (when $\alpha_{3}$ > 0, $\beta_{3}$> 0 and γ_3_ > 0), and it takes a value of ‘0’. Hence, it is otherwise assumed to increase poverty (when $\alpha_{3}$< 0, $\beta_{3}$ < 0 and γ_3_ < 0) in which case, it takes a value of ‘1’.

### 2.2 Technique of estimation

For results to be empirically compared with the literature concerning human capital development and poverty reduction, the estimation of Eqs. (1) and (2) was done using three different methods. First, the impact of human capital development on poverty reduction was estimated using the standard Ordinary Least Squares (OLS) method. Secondly, OLS estimation pools observations across cross-sections. Thirdly, by using all the variation in the data, tends to be more efficient than performing individual OLS on repeated cross-sections. However, estimating Eqs. (1) and (2) by OLS raises several concerns as it fails to account for the potential endogeneity of the explanatory variables. One immediate problem is that${\text{pov}}_{\text{it}}$ is connected with the fixed effects $\eta_{\text{i}}$in the error term, which gives rise to dynamic panel bias, according to Kaasschieter (2014) in Nickell (1981). The coefficient estimates for ${\mathrm{pov}}_{\text{it}}$is inflated by attributing a predictive power that belongs to the four selected countries fixed effects. Second, Hsiao (1986) points out that, since causality between the endogenous variable and the right-hand side variables could run in both directions, regressors may also be correlated with the disturbances. Correlation between regressors and the disturbances violates an assumption necessary for the consistency of OLS, and consequently, OLS will yield biased and inconsistent coefficient estimates. This endogeneity problem is a common problem in cross-country research and could be traced back to two generally recognised sources other than reverse causality, which are omitted variables and measurement errors.

Firstly, this study carries out the descriptive statistics and the unit root test to determine the properties of the series. Afterwards, the long-run relationship was investigated using Johansen Fisher Panel Cointegration and Fully Modified Least Square. The panel unit root and stationary test have become extremely popular and widely over the last decades when several experiments are used in software packages, the use of these test has increased significantly, hence, panel series variables have the tendency of been non-stationary at the level which may likely affect the parameter stability and consistency of the model. The most common tests in empirical research are the Levin-Lin (LL), Im-Pasaram-Shin (IPS) and the Maddala-Wu (MW). IPS test is used in association with any parametric unit root test as long as the panel is balanced and all the t-statistics for the unit root in every cross-section are identically distributed so that they will have the same variance and mean. Nevertheless, IPS is a test most often used in practice because it is simple and easy to use. Until today, most researchers have used IPS with the ADF or DF in estimating equations. This study uses balanced panel data. However, to identify the stationarity conditions of the variables, the paper uses four tests, namely: Levin, Lin & Chu, LPS, ADF-Fisher and PP- Fisher. In the presence of unit root, there is a tendency of a long-run relationship among the variables (Phillips & Hansen, 1990). Therefore, the long-run relationship among the series was tested using Johansen Fisher Panel Cointegration. Due to the presence of cointegration, the study proceeds to estimate the long-run equation using Panel Fully Modified OLS. The Panel Modified OLS gives a consistent estimate of the coefficient, helps to eliminate endogeneity and correlation in the error terms (Ramirez, 2016; Kao and Chiang, 2000).

### 2.3 Sources of data and measurement

This study focuses on 12 OPEC member countries as follows: Algeria, Angola, Congo Republic, Ecuador, Equatorial Guinea, Gabon, Republic of Iran, Kuwait, Libya, Nigeria, Saudi Arabia and Venezuela. The period of investigation is from 1980 to 2016. The variables considered in this study are poverty proxy by human development index, education proxy by expenditure on education and health proxy by spending on health. The data for poverty is obtained from World Bank, Poverty Action Lab and SEDAC-Global Distribution of Poverty. Education and health expenditure are obtained from the World Bank, Africa Economic Outlook and Organization for Economic Co-operation and Development (OECD). The study uses a balanced panel data.

## 3 Result and discussion

### 3.1 Descriptive statistics

Table 1 presents the descriptive characteristics of all the variables used in this study. The government expenditure on health and education are in log form, while the poverty rate is in normal form. The mean and median statistics of the health expenditure are 24.44087 and 24.20399, respectively. The poverty rate is 20.06635 and 11.95458 for mean and median, respectively. It was observed that the variables were positively skewness of the variable of 1.101892 and 0.767934 and their mean is significantly higher than their median, while educational expenditure is 20.61246 and 21.18389 for the mean and median respectively. The report shows that the mean is less than the median; it is a reflection of negative skewness of the variable of -1.000033. Normality test was also done using the Jarque Bera of the variables, which reaffirmed this result as reported in Table 1. According to Jarque Bera Statistics, all the variables were not normally distributed at 5 per cent level of significance; this informed the decision of logging the variables to enhance their normality and consistency behaviour, which might have been undermined as a result of non-normality. Also observed from Table 1, the standard deviation of the variables for the log of education is 2.259482, the log of health is 3.403478 and poverty rate is 17.66546; indicates a high level of volatility efficiency.

**Table 1:** Descriptive statistics of the variables used.

Variables	EDUC	HEALTH	POV
Mean	20.61246	24.44087	20.06635
Median	21.18389	24.20399	11.95458
Maximum	24.73652	33.87386	63.50000
Minimum	13.02188	19.63481	-0.055764
Std. Dev.	2.259482	3.403478	17.66546
Skewness	-1.000033	1.101892	0.767934
Kurtosis	4.282138	3.960550	2.224831
Jarque-Bera	95.95038	101.3788	54.75585
Probability	0.000000	0.000000	0.000000
Sum	8409.883	10289.61	8909.459
Sum Sq. Dev.	2077.841	4865.137	138246.4
Observations	444	444	444

### 3.2 Stationarity test: panel unit root test of the variables

Panel unit root testing emerged from time series unit testing. The major difference to time series test of a unit root is that we have to consider the asymptotic behaviour of the time series dimension T and the cross-sectional dimension N. How N and T converge to infinity is critical. If one wants to determine the asymptotic behaviour of estimators and test used for non-stationary panels through sequential limit theory, that is, no dimension is fixed, diagonal path limit. That is, N and T go to infinity along the diagonal path and the joint limit where N and T are allowed to go through infinity at the same time.

The unit root test results from Levin, Lin and Chu, Im-Pasaram-Shin, ADF-Fisher and PP- Fisher for both EDUC and HEALTH in Table 2 indicates that all the variable are not stationary at level. However, all the variables become stationary after first differencing that is; they are significant at 5 per cent except for poverty rate of -0.75771 with its IPS after first differencing is not integrated of order 1 (1) and is not significant at 5 per cent. Nonetheless, it shows that all the variables are integrated of order one that is 1 (1). Therefore, it becomes necessary to conduct the cointegration test to determine the long-run relationship among the variables. The outcome of the cointegration test justified the use of panel FMOLS (Phillips & Hansen, 1990; Asaleye et al., 2018a, Asaleye et al., 2018b, Asaleye et al., 2018c).

**Table 2:** Panel unit root test.

Variable	Method	Level	First Difference
EDUC	Levin, Lin & Chu t	0.84891	13.9956*
	LPS	2.60622	-12.9046*
	ADF_fisher	9.72371	216.378*
	PP- Fisher	10.2846	222.061*
HEALTH	Levin, Lin & Chu t	2.74398	-15.9539*
	LPS	5.49628	-16.6773*
	ADF_fisher	10.2300	246.397*
	PP- Fisher	10.4716	274.876*
POV	Levin, Lin & Chu t	-1.04141	3.95467*
	LPS	-0.62787	-0.75771
	ADF_fisher	15.0489	37.3375*
	PP- Fisher	27.5218*	23.5017*

Note: *significant at 5%.

### 3.3 Co integrating test results

The findings in Table 3 shows the Johansen Fisher Panel cointegration. The test showed that the variables were stationary at first difference; therefore, it becomes necessary to employ Johansen Fisher Panel cointegration test to provide evidence for the existence of the long-run relationship among variables in human capital development and poverty reduction across selected OPEC member countries. Evidence from the cointegration tests results in Table 3 indicates rejection of the null hypothesis of no cointegration among the variables at 5 per cent level of significance for the model specifications. The trace statistic shows that there are at least two cointegrating relationships among the variables; this implies that the variables have a long-run relationship, suggesting that there is a presence of long-run feedback effects on the short-run dynamics of the specified model.

**Table 3:** Johansen Fisher panel cointegration test.

Johansen Fisher Panel Cointegration Test				
Unrestricted Cointegration Rank Test (Trace and Maximum Eigenvalue)				
Hypothesised No. of CE(s)	Fisher Stat.* (from trace test)	Prob.	Fisher Stat.* (from the max-eigen test)	Prob.

None*	173.6	0.0000	156.9	0.0000
At most 1	48.64	0.0009	37.81	0.0193
At most 2	44.82	0.0028	44.82	0.0028

Table 4 shows the estimate of the difference in poverty rates for OPEC member countries that spend above the globally recommended annual public investment benchmarks in education is different from poverty rates of those OPEC member countries spending above the benchmarks on health. The table shows that OPEC member countries that spend above the globally recommended annual public investment benchmarks in education have achieved a high rate of reduction in poverty rate at 1.472 per cent compare to the countries spending above benchmarks on health. The countries contributing above the benchmarks on health achieved poverty rate reduction by 0.521 per cent meaning that the interaction between country spending on health meets the global benchmark spending on education reduce the poverty rate by 1.766 per cent while spending above benchmarks on health and education spending reduce poverty by 0.08 per cent. It follows the a priori expectation of the objective that when $\alpha_{3}$ > 0, $\beta_{3}$> 0 and γ_3_ > 0 and Table 4 present the estimated empirical results of the difference in poverty rates for OPEC member countries.

**Table 4:** Presentation of Estimated Empirical Results of the difference in poverty rates.

Dependent Variable: POV Observations: 444			
Variable	Coefficient	t-Statistic	Prob.
I.EDUC	-1.472515	-3.716987	0.0002
I.HEALTH	-0.521839	-0.870689	0.3846
I.EDUC*HEALTH	-1.766108	-9.840604	0.0000
I.HEALTH*EDUC	0.080557	1.650907	0.0998
R-squared	0.961888		
Adjusted R-squared	0.860139		
S.E. of regression	3.655756		
Long-run variance	8.668462		
F-statistics	70.98556		
P-value	0.0000		

## 4 Conclusion

### 4.1 Summary of findings

It is commonly believed that human capital is a crucial contributor to economic growth and development. The provision of investment in education and health has been recognised as a mediator of national development in both the developed and developing world. The availability of these services to people is one of the major ways of improving the quality of human resource because it provides the economy with excellent trained human resource required for economic growth and development. As a result, this study investigates the interactive relationship between human capital components and poverty reduction in selected OPEC member countries. It is a cross-country study combining of a panel fully modified least squares, which reports the analysis of the panel series properties of data unit root and cointegration test of 12 countries within the OPEC region. An attempt was also made to establish the conditional effects of public investment in both education and health on poverty reduction in selected OPEC member countries when public investment in education and health is above the global benchmark of 26 per cent and 5percent respectively.

The regression analysis was based on the objective of the study, and to identify the stationarity conditions of the variables, the study used four tests namely: Levin, Lin & Chu, LPS, ADF-Fisher and PP- Fisher. The unit root test results from Levin, Lin and Chu, Im-Pasaram-Shin, ADF-Fisher and PP- Fisher for both EDU and HEALTH, which indicates that not all the variables are stationary at level. However, all the variables become stationary after first differencing that is; they are significant at 5 per cent to expect for poverty rate which its IPS after first differencing is not integrated of order 1 (1) which is not statistically significant at 5 per cent. Nonetheless, it shows that all the variables are integrated of order one that is 1 (1) which therefore determine the long-run relationship among the variables. Twelve OPEC countries were focused on because of the limited availability of data. The main empirical findings are quite striking, and they suggest that the central objective of this study has been empirically explored. The results from the revealed that:

The interactive effects of the components of human capital development have a long-run impact on poverty reduction in selected OPEC member countries. According to the result, it confirms a positive and significant effect of human capital components on the reduction of poverty in this member states. From the results, the mean and median statistics of the health expenditure and poverty rate shows that the variables were positively skewed, and the mean is significantly higher than their median while the education expenditure shows that the ‘mean’ is less than the ‘median’; this reposts a reflection of negative skewness of the variables.

The result also shows that the standard deviation of the variables for the log of education, log of health and poverty rate indicates a high level of volatility efficiency. It is also observed that human capital development, government expenditure on education and health are below the minimum threshold of the global budget allocation of 26 per cent and 5percent on these sectors bringing about an increase in poverty and less economic growth in these areas. In the same vein, the study revealed that OPEC member countries spending above the global recommended annual public investment benchmarks in education had achieved a high rate of reduction in poverty rate at 1.472 per cent compared to countries contributing above the baseline in health.

The result also explains that when public investment in education is above 26 per cent global benchmark, public expenditure in health tends to have a statistically non-significant effect on the poverty rate in the selected countries, which implies that spending on health failed to achieve positive trickle-down results. Lastly, the F- statistic conducted in this study indicates that the model the independent variables jointly explained the dependent variable. However, the adjusted R- Square is high; this is because of the interactive effects. In conclusion, the result of the importance of human resource through education and health, which has a significant impact in driving the economy forward.

### 4.2 Policy recommendation and conclusion

Base on the findings of this study: it is essential to know that all the variables included in the models have a significant impact on poverty reduction. Therefore, the study recommends the following: in all the OPEC member states under investigation, the government should increase its monetary budgets on education for obtaining learning materials; ensure a conducive environment for teaching; enhance quality and informative learning scenario, which will, therefore, lead to excellent skill acquisition and self-employment. The over-dependence on oil keeps the doors locked in front of diversification strategies and causes less concentration on economic activity; therefore, countries should look into other sectors and try and increase the level of economic complexity.

The government should increase the health care facilities and motivate the health personnel with reasonable remuneration to guarantee an increase in productivity in all the various sectors of the economy. The government should also enhance the standard of education by motivating and retraining the teachers at all levels while increasing educational infrastructural facilities. In spite of the impressive growth and enormous increased performance in government expenditure among the OPEC member countries, the countries are still faced with low-level investment in human capital development which leads to a problem of the resource curse, and this tends to have less economic growth and worse development outcomes. There is, therefore, the need to link the growth attained with sound economic policies as well as the capacity to implement those policies to translate them into better performance of alleviating poverty in these OPEC member countries. Thus, since human capital is an essential key determinant of improving economic growth, the quality of human capital should be invested in education and health, improving the living standard of people and society's welfare.

## Declarations

### Author contribution statement

B.C. Olopade: Conceived and designed the experiments; Wrote the paper H. Okodua: Performed the experiments M. Oladosu: Contributed reagents, materials, analysis tools or data A. J. Asaleye: Analyzed and interpreted the data.

### Funding statement

This work was supported by Landmark University, Omu-Aran, Kwara State. Nigeria.

### Competing interest statement

The authors declare no conflict of interest.

### Additional information

No additional information is available for this paper.

## References

[bib1] Aghion P., Caroli E., García-Peñalosa C. (1999). Inequality and economic growth: the perspective of the new growth theories. J. Econ. Lit..

[bib2] ANND (2009). Facing Challenges of Poverty, Unemployment, and Inequalities in the Arab Region, Do Policy Choices of Arab Governments Still Hold after the Crisis?.

[bib3] Apergis N., Katsaiti M. (2018). Poverty and the resource curse: evidence from a global panel of countries. Res. Econ..

[bib4] Arrow K. (1973). Higher education as a filter. J. Public Econ..

[bib5] Asaleye A.J., Adama J.I., Ogunjobi J.O. (2018). Financial sector and manufacturing sector performance: evidence from Nigeria. Invest. Manag. Financ. Innov..

[bib6] Asaleye A.J., Lawal A.I., Popoola O., Alege P.O., Oyetade O.O. (2019). Financial integration, employment and wages nexus: evidence from Nigeria. Montenegrin J. Econ..

[bib7] Asaleye A.J., Popoola O., Lawal A.I., Ogundipe A., Ezenwoke O. (2018). The credit channels of monetary policy transmission: implications on output and employment in Nigeria. Banks Bank Syst..

[bib8] Asaleye A.J., Isoha L.A., Asamu F., Inegbedion H., Arisukwu O., Popoola O. (2018). Financial development, manufacturing sector and sustainability: evidence from Nigeria. J. Soc. Sci. Res..

[bib9] Attanasio O., Meghir C., Nix E., Salvati F. (2017). Human capital growth and poverty: evidence from Ethiopia and Peru. Rev. Econ. Dyn..

[bib10] Barro R.J. (1997). The Determinants of Economic Growth.

[bib11] Becker G.S. (1975). Human Capital: A Theoretical and Empirical Analysis, with Special Reference to Education. http://www.nber.org/books/beck75-1.

[bib12] Becker G.S. (1995). Human Capital and Poverty Alleviation. Human Resources Development and Operations Policy (HRO) Working Papers 14458. http://documents.worldbank.org/curated/en/121791468764735830/pdf/multi0page.pdf.

[bib13] Bhukuth A., Roumane A., Terrany B. (2018). Cooperative, human capital and poverty: a theoretical framework. Econ. Sociol..

[bib14] Blanden J., Machin S. (2004). Educational inequality and the expansion of UK higher education. Scott. J. Political Econ..

[bib15] Bloom D.E., Canning D. (2003). The health and poverty of nations: from theory to practice. J. Hum. Dev..

[bib16] Brambor T., Clark W.R., Golder M. (2006). Understanding interaction models: improving empirical analyses. Political Anal..

[bib17] Bravo-Ortega C., De Gregorio J. (2005). The Relative Richness of the Poor? Natural Resources, Human Capital, and Economic Growth. Natural Resources, Human Capital, and Economic Growth (January 2005).

[bib18] Bulte E.H., Damania R., Deacon R.T. (2005). Resource intensity, institutions, and development. World Dev..

[bib19] Burrill D.F. (2003). Modeling and Interpreting Interactions in Multiple Regression.

[bib20] Commission on Growth and Development (2008). The Growth Report: Strategies for Sustained Growth and Inclusive Development.

[bib21] Cumming D.J., Johan S., Uzuegbunam I.S. (2019). An anatomy of entrepreneurial pursuits in relation to poverty.

[bib23] Fashina O.A., Asaleye A.J., Ogunjobi J.O., Lawal A.I. (2018). Foreign aid, human capital and economic growth nexus: evidence from Nigeria. J. Int. Stud..

[bib24] Fisher A.G.B. (1946). Education and Economic Change.

[bib25] Fosu A.K. (2017). Growth, inequality, and poverty reduction in developing countries: recent global evidence. Res. Econ..

[bib26] Gamu J., Le Billon P., Spiegel S. (2015). Extractive industries and poverty: a review of recent findings and linkage mechanisms. Extr. Ind. Soc..

[bib27] Goderis B., Malone S.W. (2011). Natural resource booms and inequality: theory and Evidence. Scand. J. Econ..

[bib28] Gylfason T. (2000). Natural Resources, Education, and Economic Development, for the 15th Annual Congress of the European Economic Association, Bolzano, 30 August – 2 September 2000.

[bib29] Gylfason T., Herbertsson T., Zoega G. (1999). A mixed blessing: natural resources and economic growth. Macroecon. Dyn..

[bib30] Hsiao C. (1986). Analysis of Panel Data.

[bib31] IMF (2014). Algeria Selected Issues, International Monetary Fund (IMF) Country Report No. 14/342.

[bib69] Kaasschieter J. (2014). Remittances, economic growth, and the role of institutions and government policies. https://www.semanticscholar.org/paper/Remittances%2C-Economic-Growth%2C-and-the-Role-of-and-Kaasschieter/7a29a9adcafee53266c823ee9cd9db7c49e06576.

[bib32] Kao C., Chiang M.H., Baltagi B.H. (2000). On the estimation and inference of a cointegrated regression in panel data.

[bib33] Larionova N.I., Varlamova J.A. (2015). Analysis of human capital level and inequality interaction. Mediterr. J. Soc. Sci..

[bib34] Loayza N., Raddatz C. (2010). The composition of growth matters for poverty alleviation. J. Dev. Econ..

[bib35] Marshall A. (1920). Principles of Economics.

[bib36] Messkoub M. (2008). Economic Growth, Employment and Poverty in the Middle East and North Africa, Employment and Poverty Programme, Working Paper No. 19.

[bib37] Mincer (1991). Education and Unemployment.

[bib38] Mincer (1994). Investment in U.S Education and Training.

[bib39] Multidimensional Poverty Index Model (2016). By Duncan Green. From Poverty to Power. https://oxfamblogs.org/fp2p/tag/multidimensional-poverty/.

[bib40] Muzima J. (2018). 2018 African Economic Outlook.

[bib41] Nickell S. (1981). Biases in dynamic models with fixed effects. Econometrica.

[bib42] Ogwumike F.O., Ozughalu U.M. (2018). Empirical evidence of child poverty and deprivation in Nigeria. Child Abuse Negl..

[bib43] Oloni E., Asaleye A., Abiodun F., Adeyemi O. (2017). Inclusive growth, agriculture and employment in Nigeria. J. Environ. Manag. Tour..

[bib44] Osabuobien, Efobi (2013). Africa's money in Africa. SAJE. South Afr. J. Econ..

[bib45] Osoba A., Tella S. (2017). Human capital variables and economic growth in Nigeria: an interactive effect. EuroEconomica.

[bib46] Papyrakis E., Gerlagh R. (2004). The resource curse hypothesis and its transmission channels. J. Comp. Econ..

[bib68] Phillips P., Hansen B. (1990). Statistical inference in instrumental variables regression with I(1) processes. Rev. Econ. Stud..

[bib47] Popoola O., Asaleye A., Eluyela D. (2018). Domestic revenue mobilization and agricultural productivity: evidence from Nigeria. J. Adv. Res. Law Econ..

[bib48] Punam P. (2014). Africa new economic landscape. Brown J. World Aff..

[bib49] Ramirez M.D. (2016). A Panel Unit Root and Panel Cointegration Test of the Complementarity Hypothesis in the Mexican Case, 1960 – 2001 Center Discussion Paper No. 942.

[bib50] Ridell, Song (2011). The Impact of Education on Employment Incidence and Re-employment success: Evidence from the U.S. Labour Markets. EconPaper. Working Paper.

[bib51] Roemer J.E. (1998). Equality of Opportunity.

[bib52] Ross M.L. (2003). The natural resource curse: how wealth can make you poor. Nat. Resour. Violent Conflict.

[bib53] Sachs J.D., Warner A.M. (1997). Sources of slow growth in African economies. J. Afr. Econ..

[bib54] Sachs J.D., Warner A.M. (1995). Natural Resource Abundance and Economic Growth. NBER Working Paper, 5398.

[bib55] Santos M.E. (2009). Human Capital and the Quality of Education in a Poverty Trap Model, Oxford Poverty & Human Development Initiative (OPHI).

[bib56] Schultz T.W. (1962). Investment in Human Beings.

[bib57] Shahpari G., Davoudi P. (2014). Studying effects of human capital on income inequality in Iran, 2nd world conference on business, economics and management - WCBEM 2013. Procedia – Soc. Behav. Sci..

[bib58] Silva I., Sumarto S. (2014). Dynamics of Growth, Poverty and Human Capital: Evidence from Indonesian Sub-national Data, Munich Personal RePEc Archive (MPRA) Paper No. 65328. http://mpra.ub.uni-muenchen.de/65328/.

[bib59] Teixeira P.N. (2014). Gary Becker's early work on human capital: collaborations and distinctiveness. IZA J. Lab. Econ..

[bib60] Ucal M., Bilgin M.H. (2009). Income Inequality and FDI in Turkey: FM-OLS (Phillips-Hansen) Estimation and ARDL Approach to Cointegration, Munich Personal RePEc Archive (MPRA) Paper No. 48765. http://mpra.ub.uni-muenchen.de/48765/.

[bib61] UNESCO (2018). Government Expenditure Per Student as a Percentage of GDP Per Capita.

[bib62] WDI (2016). Poverty Headcount Ratio at National Poverty Line (Per Cent of the Population).

[bib63] WHO (2002). The World Health Report 2002 - Reducing Risks, Promoting Healthy Life.

[bib66] World Bank (2005). Introduction to poverty analysis. http://siteresources.worldbank.org/PGLP/Resources/PovertyManual.pdf.

[bib64] World Bank (2015). Poverty Forecasts, Monitoring Global Development Prospects.

[bib67] World Bank (2016). Talking on inequality, poverty and shared prosperity 2016.

